# Clonal integration facilitates the colonization of drought environments by plant invaders

**DOI:** 10.1093/aobpla/plw023

**Published:** 2016-05-06

**Authors:** Yaiza Lechuga-Lago, Marta Sixto-Ruiz, Sergio R. Roiloa, Luís González

**Affiliations:** ^1^Department of Plant Biology and Soil Science, University of Vigo, Vigo 36310, Spain; ^2^BioCost Group, Department of Animal Biology, Plant Biology and Ecology, Faculty of Sciences, Universidade da Coruña, A Coruña 15071, Spain

**Keywords:** Biomass partitioning, chlorophyll fluorescence, physiological integration, plant invasions, water stress

## Abstract

Biological invasion represents one of the main threats for biodiversity conservation at the global scale. Identifying the mechanisms underlying the process of biological invasions is a crucial objective in the prediction of scenarios of future invasions and the mitigation of their impacts. In this sense, some plant attributes might better explain the success of invasive plant species than others. Recently, clonal growth has been identified as an attribute that could contribute to the invasiveness of plants. In this experiment, we aim to determine the effect of physiological integration (one of the most striking attributes associated with clonal growth) in the performance (at morphological and physiological levels) of the aggressive invader *Carpobrotus edulis*, when occupying stressful environments. To achieve this objective we performed a greenhouse experiment in which apical ramets of *C. edulis* were water-stressed and the connection with the basal ramets was either left intact (physiological integration is allowed) or severed (physiological integration is impeded). Our results show that clonal integration allowed apical ramets to buffer drought stress in terms of photochemical activity, and as a consequence, to increase their growth in comparison with severed apical ramets. Interestingly, this increase in biomass was mainly due to the production of aboveground structures, increasing the spread along the soil surface, and consequently having important implications for the colonization success of new environments by this aggressive invader.

## Introduction

Biological invasions occur when non-indigenous species spread to areas outside their region of origin (Colautti and MacIsaac 2004; Jeschke and Strayer 2005). In this new environment their descendants proliferate, spread and persist (Mack *et al.* 2000). These invasive species have a high potential to alter the structure and function of the ecosystem (e.g. alterations of the physical, chemical and biological soil properties, nutrient cycling and plant productivity) (Levine *et al.* 2003; Otfinowski and Kenkel 2008; Chacón *et al.* 2009; Castro-Díez *et al.* 2012; Novoa *et al.* 2014; Souza-Alonso *et al.* 2015). As a consequence, invasive species are a growing threat to native biodiversity and ecosystem functions worldwide (D'Antonio and Vitousek 1992; Otfinowski and Kenkel 2008).

One of the core questions in biological invasions is to understand the biotic and abiotic mechanisms that facilitate the success of the most harmful invasive species (Levine *et al.* 2003; Blackburn *et al.* 2011; Novoa *et al.* 2012). Identifying the mechanisms underlying the process of biological invasions is a crucial objective to predict scenarios of future invasions and mitigate their impacts. Invasive plant species have a range of strategies to compete with native species (Martínez and García-Franco 2004). In this sense, some plant attributes might better explain the success of invasive species than others. Recently, clonal growth has been pointed out as an attribute that could contribute to the invasiveness of plants (Liu *et al.* 2006; Song *et al.* 2013). Clonal plants are usually dominant species in terrestrial ecosystems, and play an important role in the dynamics of many plant communities. In particular, the capacity for physiological integration (i.e. transport of resources between connected ramets) allows clonal plants to colonize successfully across a wide range of habitats (Hartnett and Bazzaz 1983; Alpert and Mooney 1986; Slade and Hutchings 1987; Alpert 1999; Saitoh *et al.* 2002; Roiloa and Retuerto 2006, 2012; Roiloa *et al.* 2014a). Although the most aggressive invasive plant species show clonal propagation, little is known about the role of clonal traits in successful invaders (but see Wang *et al.* 2008; Yu *et al.* 2009; Roiloa *et al.* 2010, 2013, 2014b, c; Song *et al.* 2013). Recently, Song *et al.* (2013) have conducted a meta-analysis highlighting the importance of traits related to clonal propagation in successful invaders. Similarly, previous experiments have studied the effect of physiological integration in the competitive ability of clonal invaders (Wang *et al.* 2008; Roiloa *et al.* 2010), demonstrated the benefit of clonal traits, as division of labour, in the propagation of aggressive invaders into heterogeneous environments (Roiloa *et al.* 2013, 2014b), or tested the impact of physiological integration in the structure of native communities (Yu *et al.* 2009). However, knowledge about the role of clonal propagation in plant invasion is still poor, and more research in necessary to fully understand the real contribution of clonal traits to invasiveness.

In this experiment, we aim to determine the effect of physiological integration in the performance (at physiological and morphological level) of the aggressive clonal invader *Carpobrotus edulis*. Particularly, we predict that physiological integration will increase the colonization capacity of this invader, especially when occupying stressful environments. To achieve this objective, we performed a greenhouse experiment in which apical ramets of *C. edulis* were water-stressed and the connection with the basal ramets was either left intact (physiological integration is allowed) or severed (physiological integration is impeded). Both in the native and invaded range, *C. edulis* frequently colonizes fore-dunes of coastal habitats. Water content is one of the most important limiting factors for plant growth in coastal sand dunes (Maun 2009). Sandy soils have high porosity and low capacity to retain water, even when rain precipitation is high. The role of physiological integration in buffering these stressful water conditions could be crucial for the successful expansion of *C. edulis* into coastal sand dunes. Coastal dunes are constantly changing environments. Additionally, these ecosystems have a high ecological value and support many threatened and endemic species (Cater 1995; Martínez 2004; Maun 2009). Plant species living in these types of ecosystems have a high degree of specialization (Wiedemann and Pickart 2004). An important part of this threat can be attributed to invasions by alien plants, as the study species *C. edulis* (Novoa *et al.* 2012).

Specifically, we aim to address the following questions: (i) Does physiological integration increase photochemical activity and growth of *C. edulis*? We expect a benefit for apical ramets derived of the support received from basal ramets. (ii) If so, are the benefits more evident when apical ramets grow under water-stressed conditions? We predict that the benefit will be more noticeable for apical ramets in stressful conditions, where support could be essential to maintain photochemical activity and growth.

## Methods

### Study species

*Carpobrotus edulis* is a succulent, perennial clonal species native to South Africa that finds suitable habitats in many coastal Mediterranean regions around the world. It was originally introduced to Southern Europe to stabilize coastal sand dunes, and as an ornamental plant in the early 20th century. Since then it has become an aggressive invader with a negative impact on the diversity of the native flora (D’Antonio and Mahall 1991; D’Antonio 1993; Wisura and Glen 1993; Traveset *et al.* 2008; Vilà *et al.* 2008). This negative impact also affects exotic species inhabiting with *C. edulis*, and remains even after *C. edulis* has been removed (Magnoli *et al.* 2013). Clonal propagation allows *C. edulis* to spread horizontally by the production of numerous ramets that remain physiologically integrated by stolon connections. This type of clonal reproduction allows a successful colonization of the surrounding area by *C. edulis*. In particular, *C. edulis* has spread to become invasive in coastal habitats, including rocky coast, cliffs and coastal sand dunes. In coastal sand dunes *C. edulis* colonizes both fore-dune and back-dune systems, occupying open sandy areas or displacing typical coastal dune flora, which in the case of NW Iberian Peninsula, include*: Euphorbia paralias, Eryngium maritimum, Calystegia soldanella, Pancratium maritimum, Otanthus maritimus, Silene littorea, Corema album* and*, Medicago marina, Honckenya peploides* or *Linaria arenaria.* These species represent a very fragile community and some of them are catalogued as endangered species, increasing the negative impact of *C. edulis* in the native habitats.

### Experimental design

Forty similarly sized clonal fragments consisting of four ramets of *C. edulis* were collected in a dune system of the O Grove peninsula (NW Spain) (42°28′22′′, N 8°51'20′′W). Clonal fragments were obtained by removing the four apical un-rooted ramets from the maternal clump. Therefore, all clonal fragments contained the first, second, third and fourth ramets from the stolon apices, which allowed us to standardize the age, size and developmental stage of the plant material used in the experiment. Sampling protocol involved the collection of each fragment from a different clump, to assure that the plants used in the experiment were not all of the same genotype.

**Figure 1.**
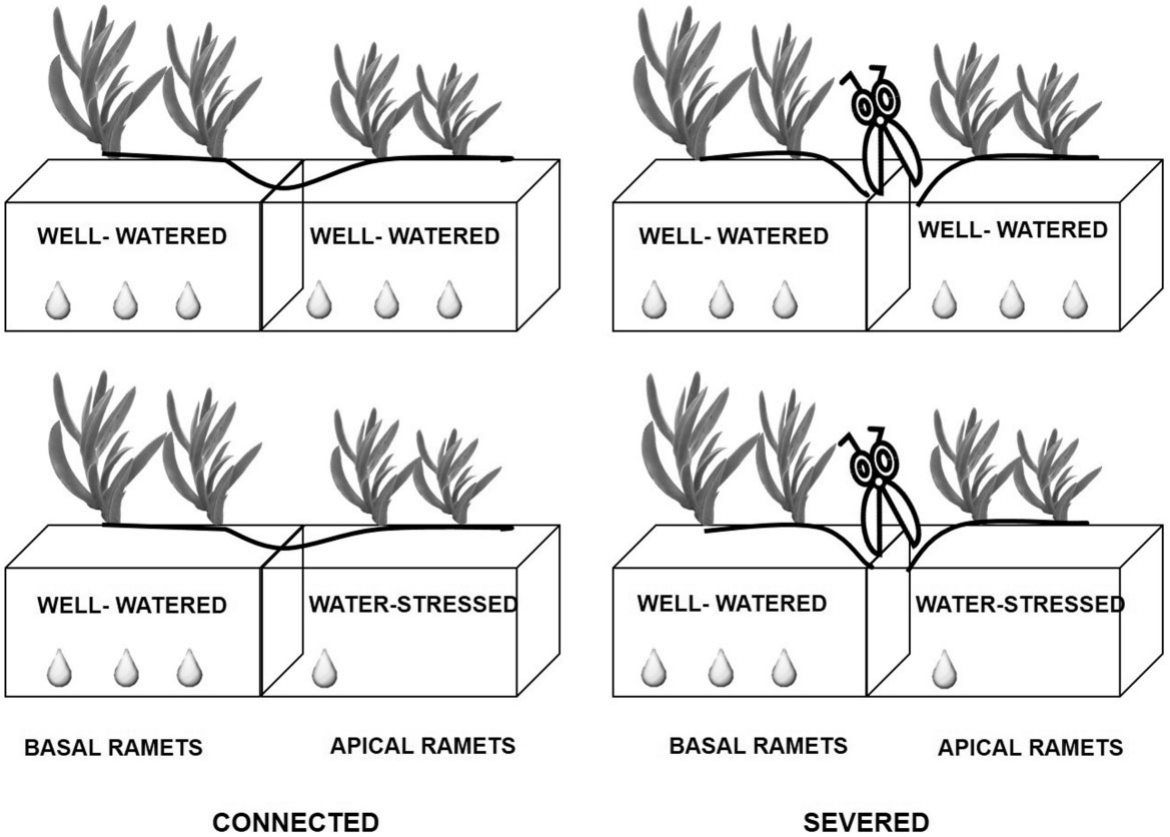
Schematic representation of the experimental treatments, with connections (connected, severed) and water availability (well-watered, water-stressed) as main factors. See text for detailed description of the experimental design.

Collected clonal fragments were individually planted in plastic containers (50 cm long × 17 cm width × 16 cm depth) filled with sand from the same dune system where the plants were collected. Plastic containers were physically divided in two patches, with the two basal and the two apical ramets of each four-ramet clonal fragment growing in each patch. The experimental design comprises of crossed factors, with connection (connected, severed) and water availability (well-watered, water-stressed) as treatments (see Fig. 1). The severed treatment was applied by cutting the stolon connection halfway between the second and third ramet from the stolon apices. Ramets in the well-watered treatment were watered regularly with as much water as necessary to maintain soil at field capacity. Ramets in the water-stressed treatment received no water at all during the experiment. Because we aimed to test the support from basal to apical ramets, basal ramets grew always in well-watered conditions, whereas the apical ramets grew either in well-watered or in water-stressed conditions. The reasons for this design were two: (i) maximize potential for sharing of resources with basal ramets growing in favourable conditions and apical ramets growing in unfavorable patches and (ii) mimic natural conditions during the invasion of *C. edulis*, where developing ramets spreads horizontally and colonize un-vegetated sand dune (with low capacity for water retention) whereas that established basal ramets have modified the soil where they grow, creating a well-watered stratum (Novoa 2012). Each treatment was replicated 10 times. The experiment was carried out for 3 months in a greenhouse at the University of Vigo (Spain).

### Measurements

In this study, we include a wide battery of biometric (total biomass and allocation ratios), physiological (photochemical efficiency determined by chlorophyll fluorescence) and biochemical (pigment and protein content) measurements that allow us to the test the hypothesized benefit of clonal integration at physiological and morphological levels. Biochemical and physiological techniques used in our study allow a rapid and accurate determination of plant health, and represent an efficient tool for stress detection. There is a direct connection between pigment content, photochemical activity and plant growth, and responses detected at biochemical and physiological levels are expected to be translated to plant growth. By using these different approaches we can obtain a whole picture of the benefit derived from clonal integration.

#### Biometric

At the end of the experiment basal and apical ramets were harvested separately, they were divided into shoot and root mass, dried at 60 °C to constant mass and weighed. For each basal and apical ramet the total mass (shoot + root mass) and the proportional biomass allocated to roots (root mass ratio, RMR = root/total mass) were calculated.

### Physiological and biochemical

#### Chlorophyll ﬂuorescence

Maximum quantum yield of photosystem II (*F*_v_/*F*_m_) was determined using a portable fluorometer (Fluorescence Monitoring System FMS 2; Hansatech Instruments, England). *F*_v_/*F*_m_ was determined as the ratio (*F*_m _−_ _*F*_0_)/*F*_m_ (see Bolhàr-Nordenkampf *et al.* 1989) where *F*_0_ and *F*_m_ are, respectively, the minimal and maximal ﬂuorescence yields of a dark-adapted sample, with all PSII reaction centres fully open. This variable was measured after a 30 min dark adaptation period, to allow PSII reaction in the centers of the leaf to be fully open. *F*_v_/*F*_m_ is a measure of the photosynthetic process associated with electron transport during the light reaction of the photosystems, and an increase in this ratio is interpreted as a reduction in the efﬁciency of excitation energy captured by open PSII reaction centers (Maxwell and Johnson 2000). On the other hand, an increase in *F*_0_ can be interpreted as reduced effectiveness of energy transport from antenna chlorophyll to reaction centers of PSII (Briantais *et al.* 1986). We also calculated the basal quantum yield of non-photochemical processes in PSII, as the ratio *F*_0_/*F*_m_. *F*_0_/*F*_m_ increases for stressed or damaged plants (Horton and Ruban 1992).

#### Pigments determination

0.2 g of fresh plant material was ground with a mortar and pestle with liquid N_2_. For the extraction of the photosynthetic pigments (Chlorophyll *a*, chlorophyll *b* and carotenoids) 2 mL of acetone was added at 80% and centrifuged at room temperature for 10 min at 4400 rpm. Finally, the supernatant was analysed spectrophotometrically at 470, 646 and 663 nm (Lightwave, WPA) (Wellburn 1994). Chlorophyll *a*, chlorophyll *b* and carotenoids concentrations were determined according to the equation proposed by Wellburn 1994, as follows: Chl*_a_* (µg mL^−^^1^) = 12.21 A_663_− 2.81 A_646_; Chl*_b_* (µg mL ^−^ ^1^) = 20.13 A_646_− 5.03 A_663_; C_(x + c)_ (µg mL ^−^ ^1^) = (1000 A_470_− 3.27 Chl*_a_*− 104 Chl*_b_*)/198. Chlorophyll content is reduced when the plants are in stress condition (Lichtenthaler *et al.* 1996). Carotenoids participate in light harvesting and are recognized as powerful antioxidants, and excited states and singlet oxygen quenchers are involved in photoprotection. Therefore high levels of carotenoids indicate that the plant is stressed (Merzlyak *et al.* 2003).

#### Total protein content

Total protein content was determined using the Bradford method (1976). 0.2 g of fresh leaves were picked and homogenized with liquid nitrogen until spraying. The samples were centrifuged at 4 °C for 20 min at 16 000 g. Total protein content in leaves was determined spectrophotometrically at 595 nm, using bovine serum albumin as a standard reference. Low levels of protein indicate that the plant is in stress (Dhindsa and Bewley 1977).

### Statistical analyses

Prior to analyses, the data were checked for normality using a Kolmogorov–Smirnov test and for homoscedasticity using a Levene test. To meet with the requirements of normality and homoscedasticity for ANOVA, RMR in basal ramets and chl*a*, chl*b* and carotenoids were log-transformed, and *F*_0_, *F*_0_/*F*_m_ and *F*_v_/*F*_m_ were reverse-transformed in apical ramets. We used two-way analysis of variance (ANOVA) with ‘connection’ and ‘water’ as main factors to analyse differences between the treatments for all the variables studied. Separate analyses were performed for basal and apical ramets. Signiﬁcance level was set at *P* *<*0.05. Statistical tests were performed with SPSS Statistics 19.0 (IBM, Armonk, New York, USA).

## Results

### Biometric

**Figure 2.**
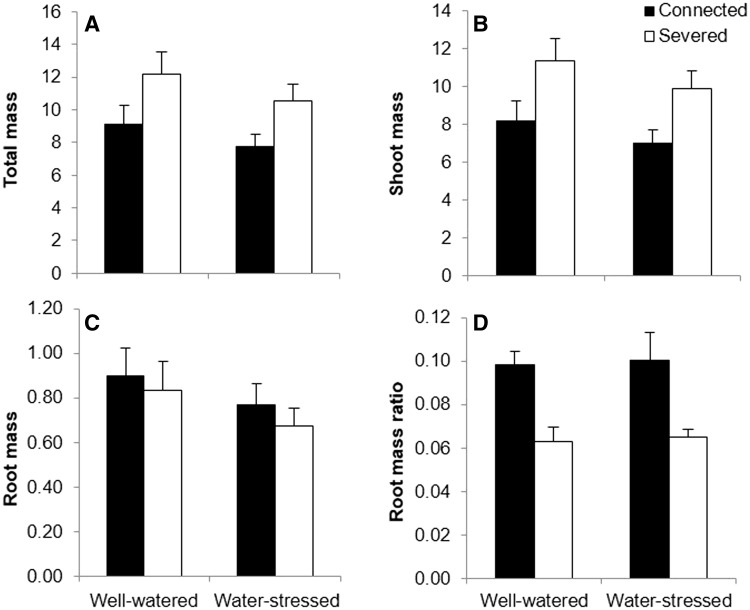
Mean (±s.e) total mass (A), shoot mass (B), root mass (C) and RMR (D) of connected (filled bars) and severed (empty bars) basal ramets in the water-stressed and well-watered treatments. See Table 1 for ANOVA parameters.

**Figure 3.**
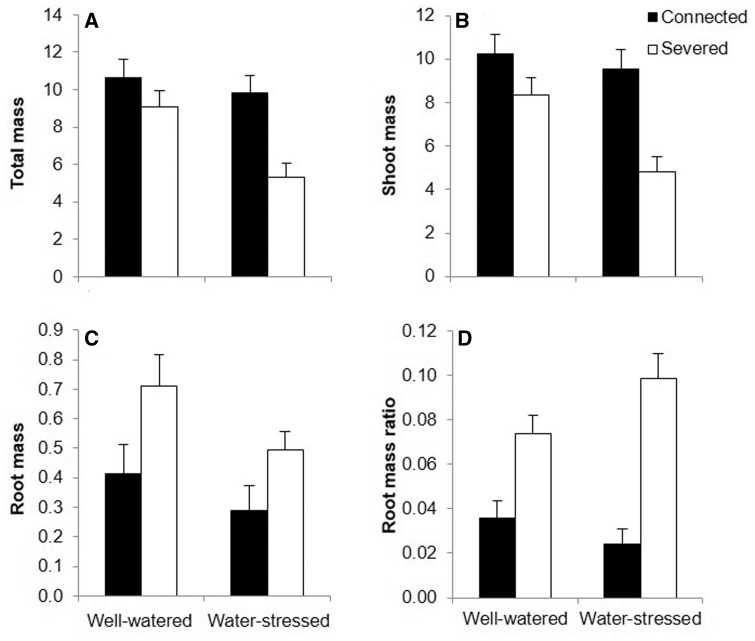
Mean (±s.e) total mass (A), shoot mass (B), root mass (C) and RMR (D) of connected (filled bars) and severed (empty bars) apical ramets in the water-stressed and well-watered treatments. See Table 1 for ANOVA parameters.

Connection significantly affected shoot mass, total mass and root mass ratio in basal ramets (Table 1). Both total and shoot mass were significantly higher for basal ramets in the severed treatment in comparison with basal connected ramets (Fig. 2A and B). However, connection significantly increased the proportional biomass allocated to root (determined by the RMR) by basal ramets (Fig. 2D). The effect of water and the interactive effect of connection by water in basal ramets were not statistically significant for any of the variables measured (Table 1). In apical ramets disconnection significantly reduced the shoot and total mass, and increased the root mass and the proportional mass allocated to roots (RMR) (Table 1 and Fig. 3). Water stress affected the total and shoot mass of apical ramets significantly, leading to a reduction in both variables (Table 1 and Fig. 3A and B). However, root mass or the RMR were not significantly affected by water treatment (Table 1 and Fig. 3C and D). Results showed a significant interaction of connection by water for the biomass allocated to roots in apical ramets (Table 1). In this sense, connection significantly reduced the proportional biomass allocated to roots by apical ramets, and this reduction was especially evident when apical ramets grew in water-stressed conditions (Fig. 3D).

**Table 1.** Results of two-way analyses of variance (ANOVA) for analyses of differences in shoot mass, root mass, total mass and RMR to examine the effects of ‘connection’ and ‘water’ for basal and apical ramets.

Effect	Total mass			Shoot mass			Root mass			RMR		
	df	*F*	P	df	*F*	P	df	*F*	P	df	*F*	P
***Basal ramets***												
Connection	1	7.11	**0.011**	1	8.93	**0.005**	1	0.55	0.46	1	17.56	**<0.001**
Water	1	1.84	0.18	1	1.79	0.19	1	1.70	0.20	1	0.07	0.80
Connection x water	1	0.02	0.89	1	0.02	0.89	1	0.01	0.90	1	0.37	0.55
Error	36			36			36			36		
***Apical ramets***												
Connection	1	12.05	**0.001**	1	16.48	**0.000**	1	8.00	**0.008**	1	42.84	**<0.001**
Water	1	6.77	**0.013**	1	6.79	**0.013**	1	3.62	0.06	1	0.63	0.43
Connection × water	1	2.79	0.10	1	3.06	0.09	1	0.25	0.62	1	4.52	**0.040**
Error	36			36			36			36		

Values with *P < *0.05 are in boldface. See Figs 2 and 3 for data.

### Physiological and biochemical

#### Chlorophyll ﬂuorescence

**Figure 4.**
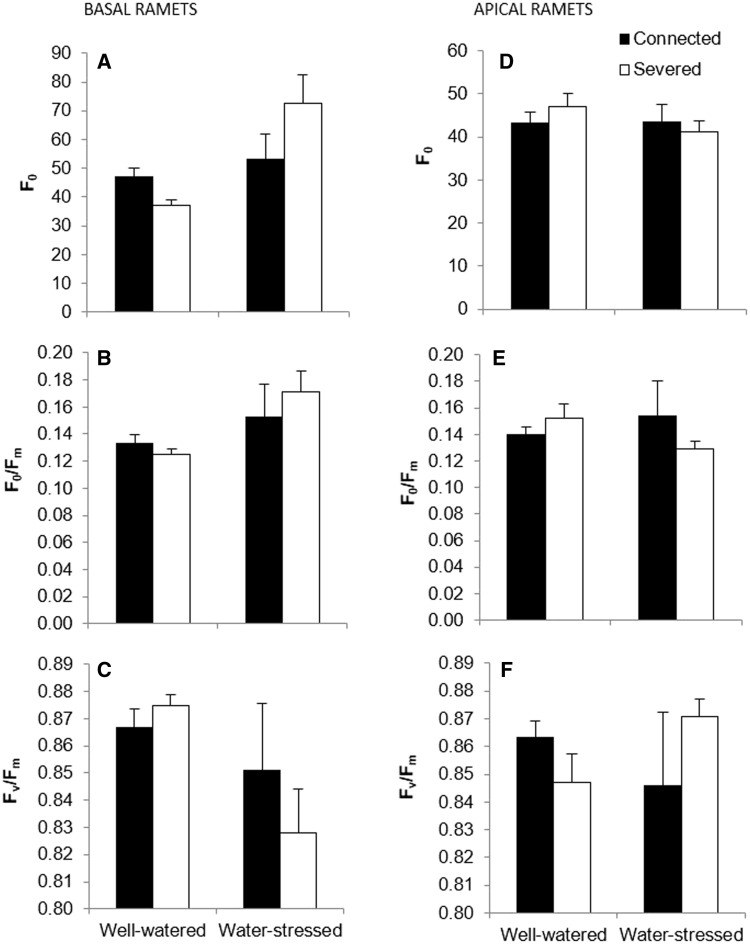
Mean (±s.e) *F*_0_ (A), *F*_0_/*F*_m_ (B) and *F*_v_/*F*_m_ (C) of basal ramets and *F*_0_ (D), *F*_0_/*F*_m_ (E) and *F*_v_/*F*_m_ (F) of apical ramets of connected (filled bars) and severed basal (empty bars) ramets in the water-stressed and well-watered treatments. See Table 2 for ANOVA parameters.

For basal ramets we did not detect any significant effects of the different treatments for the fluorescence parameters (Fig. 4 and Table 2). In apical ramets water treatment significantly affected *F*_0_ and *F*_0_/*F*_m_ (Table 2). Our results showed a significant increase both for *F*_0_ and *F*_0_/*F*_m_ (Fig. 4D and E) in the water-stressed treatment. The interaction of connection by water significantly affected *F*_0_ (Table 2). We observed a significant increase of *F*_0_ due to water deprivation, and this increase was especially evident in the severed treatment. In other words, the negative effect of the water-stressed treatment (reported by the increase *F*_0_) was buffered by the effect of the connection (i.e. physiological integration) (Fig. 4D). We did not detect significant effects of the different treatments on *F*_v_/*F*_m_ (Table 2).

**Table 2.** Results of two-way analyses of variance (ANOVA) for analyses of differences in *F*_0_, *F*_0_/*F*_m_, *F*_v_/*F*_m_ to examine the effects of ‘connection’ and ‘water’ for basal and apical ramets.

Effect	F0			F0/Fm			Fv/Fm		
	df	*F*	P	df	*F*	P	df	*F*	P
***Basal ramets***									
Connection	1	0.04	0.83	1	0.18	0.67	1	0.09	0.77
Water	1	0.81	0.37	1	0.11	0.74	1	0.04	0.84
Connection × water	1	1.00	0.32	1	1.59	0.22	1	1.94	0.17
Error	36			36			36		
***Apical ramets***									
Connection	1	0.01	0.91	1	0.12	0.73	1	0.08	0.78
Water	1	10.02	**0.003**	1	4.75	**0.036**	1	3.84	0.06
Connection × water	1	5.95	**0.020**	1	1.86	0.18	1	0.51	0.48
Error	36			36			36		

Values with *P *< 0.05 are in boldface. See Fig. 4 for data.

Pigments and protein content: In both basal and apical ramets we did not detect significant effects of the different treatments in any of the variables studied [see **Supporting Information**].

## Discussion

The results supported the proposed hypothesis of a benefit from clonal integration for apical ramets, especially for those growing in the unfavorable conditions imposed by water limitation. Specifically, we found a benefit of integration in terms of photochemical activity, and this benefit was more important for apical ramets growing in water-stressed conditions. Thus, the negative impact of water deprivation on the photochemical activity of apical ramets, determined by the increase of the minimal ﬂuorescence yield *F*_0_ (Briantais *et al.* 1986) was alleviated by the connection to basal ramets growing in well-watered conditions. Similarly, our results showed a reduction in total mass of water-stressed apical ramets, but this reduction was buffered by the connection to basal ramets. That is, support from basal ramets (i.e. clonal integration) mitigates the negative impact of water deprivation, allowing apical ramets to maintain photochemical activity levels similar to no-stress conditions and therefore allowing the growth and expansion of this invader in spite of the stressful conditions. *Carpobrotus* *edulis* invades coastal sand dunes along all the Mediterranean areas in the world (D’Antonio 2006). These habitats, which contain fragile and frequently endangered plant species, are characterized by stressful conditions, including low water availability due to the low retention capacity of the soil (de Jong 1979; Martínez *et al.* 2002). In these conditions, the capacity for physiological integration confers an advantage to *C. edulis* that could contribute to the successful invasion of coastal sand dunes (Roiloa et al. 2014c). Our results confirm previous studies in showing the benefits of integration for many other clonal plant species and under a wide variety of situations (e.g. Hartnett and Bazzaz 1983; Slade and Hutchings 1987; Yu *et al.* 2004; Roiloa and Retuerto 2006; Wang *et al.* 2008; Roiloa *et al.* 2014a). Interestingly, a recent field study reported benefits in terms of growth for *C. edulis* when invading a coastal sand dune (Roiloa *et al.* 2010). We report similar results, with a benefit of integration for apical ramets in terms of biomass production, but in addition we demonstrate that the benefit is also produced at physiological level. We found a benefit of integration in terms of photochemical activity, and interestingly our results demonstrate that benefits detected at physiological level can be transferred to growth. The chlorophyll fluorescence parameters are a sensitive indicator of plant photosynthetic performance, and as a consequence can be used for early detection of plant fitness (Maxwell and Johnson 2000). This benefit at the physiological level was especially evident in the apical ramets growing in water-stressed conditions. Drought conditions often occur in sand dune systems, and therefore our results indicate that clonal integration would contribute to the expansion of this invader in water-stressed conditions, with the consequent implications in the understanding of the invasiveness of *C. edulis* in sand dune systems.

Our results also demonstrate that clonal integration modified biomass partitioning both for basal and apical ramets. Morphological plasticity allows plants to cope with the variety of environmental situations that they encounter in natural habitats (Valladares *et al.* 2007; Mommer *et al.* 2011). In this sense plants adjust allocation between above and below-ground structures to optimize the acquisition of resources. Therefore, the optimal partitioning theory predicts that plants will increase the proportional biomass allocation to the structures responsible of acquiring the most limited resources (Thornley 1972; Bloom *et al.* 1985). In the case of clonal plants, local conditions experienced by modules can result in changes in biomass partitioning of the other members of the system. In other words, clonal integration can conduct to non-local responses in connected ramets (de Kroon *et al.* 2005, 2009; Roiloa and Hutchings 2012, 2013). Our results showed that integration significantly increased the proportional biomass allocated to roots by basal ramets. We interpreted this result as a compensatory response from established basal ramets to support developing apical ramets. Previous studies have reported that transport of resources from ramets in favourable conditions to developing ramets in stressful patches can increase the overall performance of the whole clone (Hartnett and Bazzaz 1983; Salzman and Parker 1985; Wijesinghe and Handel 1994; Roiloa and Hutchings 2012). On the other hand, we detected a significant effect of integration on the biomass allocated to roots by apical ramets. Interestingly, this effect was dependent on the availability of water experienced by the apical ramets. Thus, integration induced a non-local response in apical ramets, especially those in water-stressed conditions, significantly reducing the investment in root production. These changes in biomass partitioning would promote more extensive aboveground lateral growth of the apical ramets, with the consequent implications for the expansion of this aggressive invader.

Both the increase of the biomass allocated to roots by connected basal ramets in well-watered conditions, and the decrease of the biomass allocated to roots by connected apical ramets in water-stressed conditions, indicate that clonal integration modifies the predictions of the optimal partitioning theory (a specialization to the acquisition of the most limited resource) (Thornley 1972; Bloom *et al.* 1985). However, this change in biomass partitioning mediated by integration agrees with the division of labour theory in clonal plants. Division of labour is defined as the capacity for specialization to acquire locally abundant resources, managing to benefit the whole clone after reciprocal resource sharing between the connected ramets (Friedman and Alpert 1991; Alpert and Stuefer 1997; Hutchings and Wijesinghe 1997; Stuefer 1998). In spite of the benefits derived from division of labour for clonal plant propagation, only recently have studies been conducted to determine the contribution of ramets specialization for the invasiveness of clonal plants (Roiloa *et al.* 2013; Roiloa *et al.* 2014b, c). Division of labour has also recently been described for *C. edulis*, under homogeneous conditions (developmentally induced division of labour) (Roiloa *et al.* 2013), and also in environments with negative spatial covariance in the distribution of nutrients and light (environmental-induced division of labour) (Roiloa *et al.* 2014b). However, as far as we know, this is the first study reporting capacity of division of labour for *C. edulis* in a heterogeneous environment with favourable (well-watered) and unfavourable (water-stressed) patches.

## Conclusions

Our study reports benefits of clonal integration at morphological and physiological levels for the clonal invader *C. edulis*, specifically when colonizing water-stressed environments. Our results show that clonal integration allowed apical ramets to buffer drought stress in terms of photochemical activity, and as a consequence to increase their growth in comparison with severed ramets. Interestingly, this increase in biomass was mainly allocated to producing aboveground structures, increasing the spread along the soil surface, and consequently having important implications for the colonization success of new environments by this aggressive invader. Effects of clonal integration on physiological, growth and biomass partitioning could report a benefit to clonal plants for the colonization of stressful environments, and therefore should be considered as a plausible mechanism to explain the success of clonal invaders. Thus, our results indicate that physiological integration could be considered an important factor in the expansion of the invader *C. edulis*. However, to understand the real contribution of clonality to plant invasions, further research to test for differences in capacity for physiological integration between invasive and non-invasive clonal species is essential. Determining the contribution of clonal traits in the expansion of exotic plants provides basic information on the ecological processes behind the process of invasions, and consequently could be particularly important helping to predict future scenarios of invasions and for management practices.

## Sources of Funding

Financial support for this study was provided by the Spanish Ministry of Economy and Competitiveness (projects Ref. CGL2013-48885- C2-1-R, awarded to L.G. and Ref. CGL2013-44519-R, awarded to S.R.R.). European Regional Development Fund (ERDF) co-financed these projects. This is a contribution from the Alien Species Network (Ref. R2014/036 – Xunta de Galicia, Autonomous Government of Galicia).

## Contributions by Authors

L.G. and S.R.-R. were involved in designing the experiment. Y.L.-L., M.S.-S., S.R.-R. and L.G. in field samples collection and set up the experiment. Y.L.-L., M.S.-S. and L.G. in experiment maintenance and data collection. Y.L.-L., S.R.-R. and L.G. in statistical analysis, manuscript preparation and submission.

## Conflicts of Interest Statement

None declared.

## Supplementary Material

Supplementary Data

## Supporting Information

The following additional information is available in the online version of this article —

**Table S3.** Results of two-way analyses of variance (ANOVA) for analyses of differences in chl*a*, chl*b*, chl*a*/chl*b*, carotenoids and protein content to examine the effects of “connection” and water for basal and apical ramets. Values of *P* < *0.05* are in boldface. See Fig. 5 and Fig. 6 for data.

**Figure S5.** Mean (±s.e) chl*a* (A), chl*b* (B), chl*a*/chl*b* (C), carotenoids (D) and protein content (E) of connected (filled bars) and severed basal (empty bars) ramets in the water stressed and well-watered treatments. See Table 3 for ANOVA parameters.

**Figure S6.** Mean (±s.e) chl*a* (A), chl*b* (B), chl*a*/chl*b* (C), carotenoids (D) and protein content (E) of connected (filled bars) and severed (empty bars) apical ramets in the water stressed and well-watered treatments. See Table 3 for ANOVA parameters.
