# Supplementary material for: Clonal integration facilitates the colonization of drought environments by plant invaders
Source: AoB Plants. 2016 May 6;8:plw023. doi: 10.1093/aobpla/plw023 (PMC4925925; doi:10.1093/aobpla/plw023)
Supplement: Supplementary Data [file supp_8_plw023_index.html]

Clonal integration facilitates the colonization of drought environments by plant invaders — Clonal integration facilitates the colonization of drought environments by plant invaders — Supplementary Data 

# Clonal integration facilitates the colonization of drought environments by plant invaders

## Supplementary Data

files

- Supplementary Data - jpg file
- Supplementary Data - doc file
- Supplementary Data - jpg file
