# Supplementary figures and images for: Clonal integration facilitates the colonization of drought environments by plant invaders
Source: AoB Plants. 2016 May 6;8:plw023. doi: 10.1093/aobpla/plw023 (PMC4925925; doi:10.1093/aobpla/plw023)

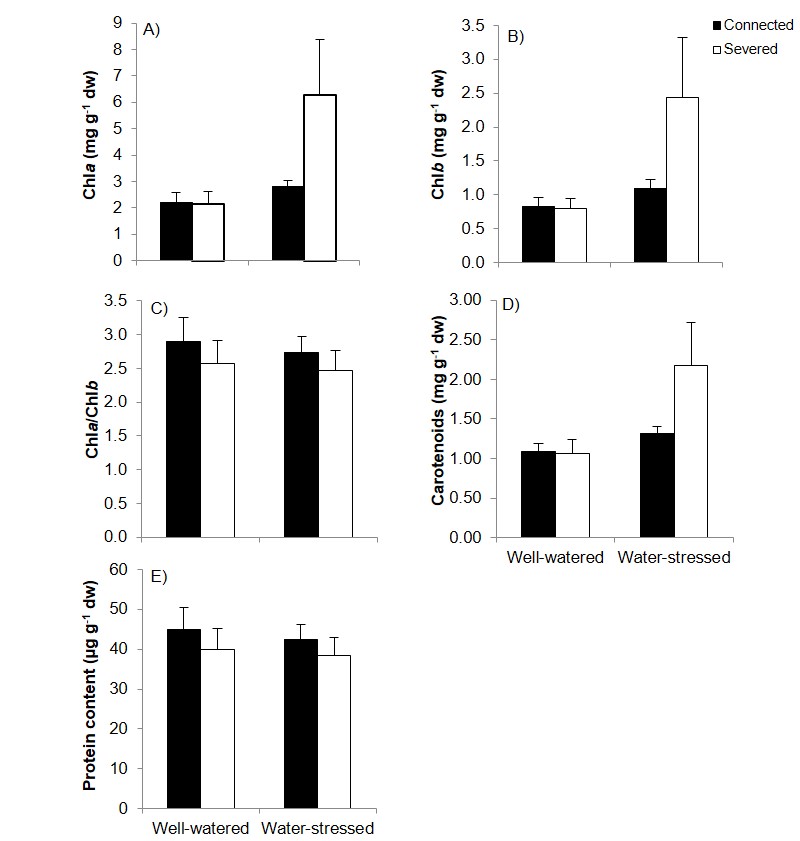

Supplement: Supplementary Data [file supp_plw023_aobplants-15342-s04.jpg]

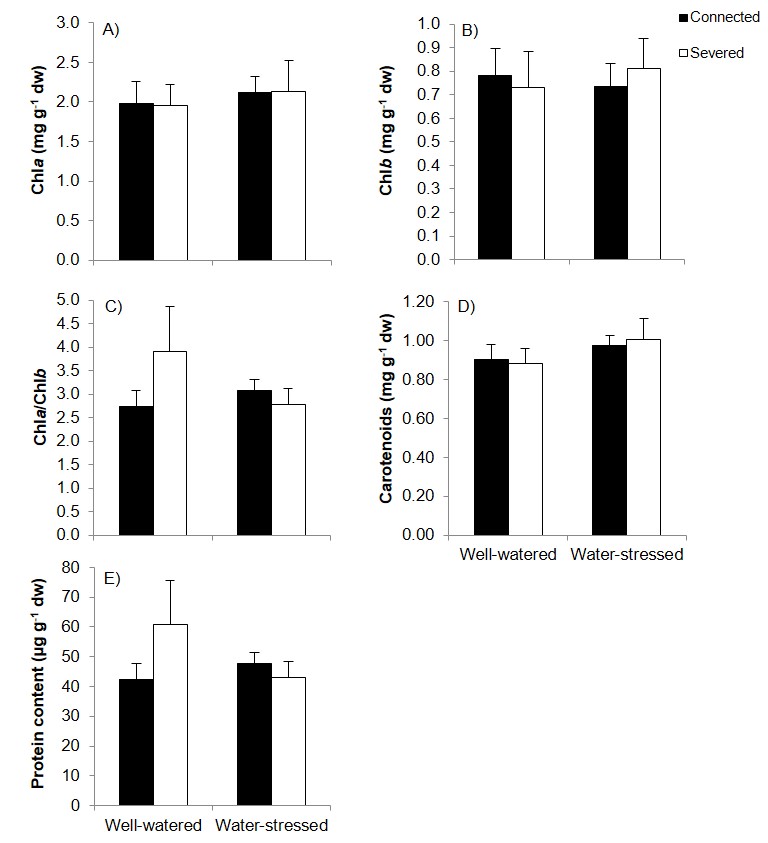

Supplement: Supplementary Data [file supp_plw023_aobplants-15342-s03.jpg]
