# Supplementary material for: Clonal integration facilitates the colonization of drought environments by plant invaders
Source: AoB Plants. 2016 May 6;8:plw023. doi: 10.1093/aobpla/plw023 (PMC4925925; doi:10.1093/aobpla/plw023)
Supplement: Supplementary Data [file supp_plw023_aobplants-15342-s02.doc]

|  | **Chl*a*** | | |  | **Chl*b*** | | |  | **Chl*a*/Chl*b*** | | |  | **Carotenoids** | | |  | **Protein content** | | |
| --- | --- | --- | --- | --- | --- | --- | --- | --- | --- | --- | --- | --- | --- | --- | --- | --- | --- | --- | --- |
| Effect | df | *F* | *P* |  | df | *F* | *P* |  | df | *F* | *P* |  | df | *F* | *P* |  | df | *F* | *P* |
| ***Basal ramets*** |  |  |  |  |  |  |  |  |  |  |  |  |  |  |  |  |  |  |  |
| Connection | 1 | 0.00 | 0.98 |  | 1 | 0.01 | 0.92 |  | 1 | 0.66 | 0.42 |  | 1 | 0.00 | 0.96 |  | 1 | 0.66 | 0.42 |
| Water | 1 | 0.26 | 0.62 |  | 1 | 0.02 | 0.89 |  | 1 | 0.51 | 0.48 |  | 1 | 1.53 | 0.22 |  | 1 | 0.51 | 0.48 |
| Connection x Water | 1 | 0.00 | 0.96 |  | 1 | 0.25 | 0.62 |  | 1 | 1.81 | 0.19 |  | 1 | 0.10 | 0.76 |  | 1 | 1.81 | 0.19 |
| Error | 36 |  |  |  | 36 |  |  |  | 36 |  |  |  | 36 |  |  |  | 36 |  |  |
| ***Apical ramets*** |  |  |  |  |  |  |  |  |  |  |  |  |  |  |  |  |  |  |  |
| Connection | 1 | 0.17 | 0.68 |  | 1 | 0.06 | 0.81 |  | 1 | 0.85 | 0.36 |  | 1 | 0.22 | 0.64 |  | 1 | 0.85 | 0.36 |
| Water | 1 | 0.35 | 0.56 |  | 1 | 0.45 | 0.50 |  | 1 | 0.16 | 0.69 |  | 1 | 0.07 | 0.79 |  | 1 | 0.16 | 0.69 |
| Connection x Water | 1 | 0.02 | 0.90 |  | 1 | 0.04 | 0.84 |  | 1 | 0.01 | 0.92 |  | 1 | 0.08 | 0.78 |  | 1 | 0.01 | 0.92 |
| Error | 36 |  |  |  | 36 |  |  |  | 36 |  |  |  | 36 |  |  |  | 36 |  |  |

**Table 3.** Results of two-way analyses of variance (ANOVA) for analyses of differences in chl*a*, chl*b*, chl*a*/chl*b*, carotenoids and protein content to examine the effects of “connection” and water for basal and apical ramets. Values of *P < 0.05* are in boldface. See Fig. 5 and Fig. 6 for data.
